# Dose of Retroviral Infection Determines Induction of Antiviral NK Cell Responses

**DOI:** 10.1128/JVI.01122-17

**Published:** 2017-10-27

**Authors:** Elisabeth Littwitz-Salomon, Simone Schimmer, Ulf Dittmer

**Affiliations:** Institute for Virology of the University Hospital Essen, University of Duisburg-Essen, Essen, Germany; Ulm University Medical Center

**Keywords:** natural killer cells, Friend retrovirus, antiviral activity, virus dose, interleukins

## Abstract

Natural killer (NK) cells are part of the innate immune system and recognize virus-infected cells as well as tumor cells. Conflicting data about the beneficial or even detrimental role of NK cells in different infectious diseases have been described previously. While the type of pathogen strongly influences NK cell functionality, less is known about how the infection dose influences the quality of a NK cell response against retroviruses. In this study, we used the well-established Friend retrovirus (FV) mouse model to investigate the impact of virus dose on the induction of antiviral NK cell functions. High-dose virus inoculation increased initial virus replication compared to that with medium- or low-dose viral challenge and significantly improved NK cell activation. Antiviral NK cell activity, including *in vivo* cytotoxicity toward infected target cells, was also enhanced by high-dose virus infection. NK cell activation following high-dose viral challenge was likely mediated by activated dendritic cells (DCs) and macrophages and the NK cell-stimulating cytokines interleukin 15 (IL-15) and IL-18. Neutralization of these cytokines decreased NK cell functions and increased viral loads, whereas IL-15 and IL-18 therapy improved NK cell activity. Here we demonstrate that virus dose positively correlates with antiviral NK cell activity and function, which are at least partly driven by IL-15 and IL-18. Our results suggest that NK cell activity may be therapeutically enhanced by administering IL-15 and IL-18 in virus infections that inadequately activate NK cells.

**IMPORTANCE** In infections with retroviruses, like HIV and FV infection of mice, NK cells clearly mediate antiviral activities, but they are usually not sufficient to prevent severe pathology. Here we show that the initial infection dose impacts the induction of an antiviral NK cell response during an acute retroviral infection, which had not investigated before. High-dose infection resulted in a strong NK cell functionality, whereas no antiviral activities were detected after low- or medium-dose infection. Interestingly, DCs and macrophages were highly activated after high-dose FV challenge, which corresponded with increased levels of NK cell-stimulating cytokines IL-15 and IL-18. IL-15 and IL-18 neutralization decreased NK cell functions, whereas IL-15 and IL-18 therapy improved NK cell activity. Here we show the importance of cytokines for NK cell activation in retroviral infections; our findings suggest that immunotherapy combining the well-tolerated cytokines IL-15 and IL-18 might be an interesting approach for antiretroviral treatment.

## INTRODUCTION

NK cells belong to the innate arm of the immune system and provide an early defense against viruses and transformed cells. NK cells use a complex array of germ line-encoded receptors and do not need clonal expansion or receptor rearrangement to initiate a response (1, 2). They can discriminate target cells from healthy cells due to expression of inhibitory major histocompatibility complex class I (MHC-I)-specific receptors (3, 4). “Missing self,” which means the absence of MHC-I molecules on target cells, or the expression of ligands for their activating receptors results in activation of NK cells (4). Activated NK cells produce a wide range of cytokines, such as gamma interferon (IFN-γ), tumor necrosis factor alpha (TNF-α), and granulocyte-macrophage colony-stimulating factor (GM-CSF), which shape innate and adaptive immunity (5). Besides cytokine production, activated NK cells can also lyse target cells by releasing cytotoxic granules that contain perforin and granzymes. Granules are exocytosed into an immunological synapse that is formed between NK cells and target cells (6). Additionally, activated NK cells express on their surface the death receptor ligands TNF-related apoptosis-inducing ligand (TRAIL) and Fas ligand (FasL), which induce apoptosis after binding to death receptors on target cells (7, 8). Effective production of antiviral molecules by NK cells requires more than a single signal from activating receptors; it also depends on secondary signals, like costimulatory molecules and cytokines (9, 10). Dendritic cells (DCs) are well-known interaction partners of NK cells that trigger proliferation, survival, and cytotoxicity of NK cells via cell-to-cell contacts and cytokine release (11–16). Interestingly, the cross talk between NK cells and DCs is bidirectional, since NK cells are able to activate DCs, restrict maturation, and lyse immature DC (12, 14, 17, 18). During infections, DCs release a variety of cytokines, such as type I IFNs, interleukin 12 (IL-12), IL-15, and IL-18, that strongly influence NK cell responses (12, 19, 20). The population of DCs can be classified into conventional DCs (cDCs) and nonconventional DCs, including the subset of plasmacytoid DCs (pDCs) (21). Upon most viral infections, cDCs produce IL-12, IL-15, and IL-18 (22–24), whereas pDCs rapidly produce large amounts of type I IFNs and also IL-12 (25–27). Similarly to DC, macrophages are also able to release the NK cell-stimulating cytokines IL-12, IL-15, and IL-18, helping NK cells to attack their target cells (28).

Numerous studies have described the involvement of NK cells in the control of viral infections (5, 10, 29, 30), but detrimental effects of NK cells on infections with viruses have also been described (31, 32). During influenza virus and lymphocytic choriomeningitis virus (LCMV) infections, the outcome of NK cell activity greatly depends on the initial infection dose. High-dose influenza virus infection results in NK cell-mediated pathology, while NK cells are beneficial at medium viral exposure (33). In contrast, during infection with a high dose of LCMV, NK cells prevent lethal immunopathology while allowing the establishment of viral persistence, whereas they do not interfere with T cell-induced mortality after medium-dose LCMV inoculation (34). Thus, this previous study (34) focused on the immunoregulatory function of NK cells on T cell responses but not on their direct antiviral activity. We were interested in how the initial infection dose impacts the induction of an antiviral NK cell response during an acute retroviral infection, which has not been investigated so far. For this study, we used the well-established Friend retrovirus (FV) mouse model, which allows the analysis of acute antiviral immune responses and the *in vivo* modulation of several immune cell populations (35–43). The FV complex consists of the nonpathogenic but replication-competent Friend murine leukemia virus (F-MuLV) and spleen focus-forming virus (SFFV), which is responsible for pathogenesis but is replication defective (44). Depending on the mouse strains, susceptible mice develop severe splenomegaly and subsequent erythroleukemia, whereas resistant mice, such as C57BL/6 mice, which were used in this study, are protected from leukemia due to genetic resistance factors and their potent immune responses. However, resistant mice also develop persistent infection after FV inoculation (44, 45). The basic antiretroviral immune responses were identified in the FV mouse model, which are quite comparable to results for HIV-infected humans (39, 46–49). NK cells become activated and show antiviral functions during acute infection with FV or HIV-1 (37, 50, 51), although FV infection with standard doses of virus resulted in only weak NK cell responses (41). Similar to the case with chronic HIV infection, antiviral NK cell functions were impaired during the later phase of FV infection (37, 52). While there are several studies on NK cells in retrovirus infections, the influence of initial viral loads on the induction of antiviral NK cell responses has not yet been elucidated. To address this issue, we explored the impact of FV infection dose on NK cell functions during acute retroviral infection.

High-dose infection resulted in strong activation, cytokine production, and cytotoxicity of NK cells, whereas NK cell responses after low- or medium-dose infection were comparable to responses in naive mice. DCs and macrophages were highly activated after high-dose FV challenge, which correlated with increased cytokine levels of the NK cell-stimulatory cytokines IL-15 and IL-18. Our data reveal an intriguing correlation of retroviral infection levels with the induction of potent NK cell responses and suggest that therapeutic manipulation of NK cells by cytokines might be a possible approach for the treatment of virus infections that inadequately activate NK cells.

## RESULTS

### Different kinetics of viral replication after medium- and high-dose FV infection.

**FIG 1:**
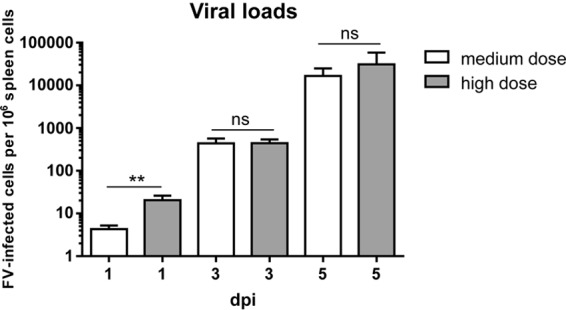
Kinetics of viral loads after medium- and high-dose FV infections. C57BL/6 mice were infected with a medium dose (20,000 SFFU) or high dose (40,000 SFFU) of FV. Viral loads were analyzed in the spleen at 1, 3, and 5 dpi by infectious-center assay. Statistically significant differences between groups were analyzed by the Mann-Whitney test (**, *P* < 0.01). At least four animals per group were examined. The experiments were repeated at least twice, with comparable results. ns, not significant.

Viral dissemination and the clinical outcome of viral infections greatly depend on various factors, such as infection routes, virus isolates, and infection doses (53–56). It was previously published that functions of immune cells were influenced by various virus inoculum doses, but results were inconsistent for different virus species (33, 34, 55). Studies on the impact of the initial retroviral infection dose on the NK cell immunity have not been performed so far. For the investigation of acute FV infection in mice of the C57BL/6 background, we routinely apply the FV complex intravenously at the medium dose of 20,000 spleen focus-forming units (SFFU) of lactate dehydrogenase-elevating virus-free FV stock (36, 39, 41, 57–59). In this study, we additionally infected mice with 40,000 SFFU (high dose) and analyzed viral loads at 1, 3, and 5 days postinfection (dpi) to monitor the initial kinetics of an acute FV infection (Fig. 1). The term spleen focus-forming units describes the titer of infectious virus particles that induce a focus of proliferating cells on a mouse spleen. Thus, it is a direct measure for virus particles that are pathogenic *in vivo*, and a 2-fold difference in SFFU titer is quite significant for viral pathogenesis. At 1 dpi we detected significantly higher viral loads after infection with a high virus dose than after the medium dose. Virus loads increased at day 3 and again at day 5 postinfection, but the difference between the groups of mice receiving different viral inoculation doses was not observed anymore. These data imply that the initial infection dose influences only the very early kinetics of viral replication, but the effect on the NK cell immunity was unknown.

### NK cells display a highly activated, maturated effector phenotype after retroviral infection with high viral doses.

**FIG 2:**
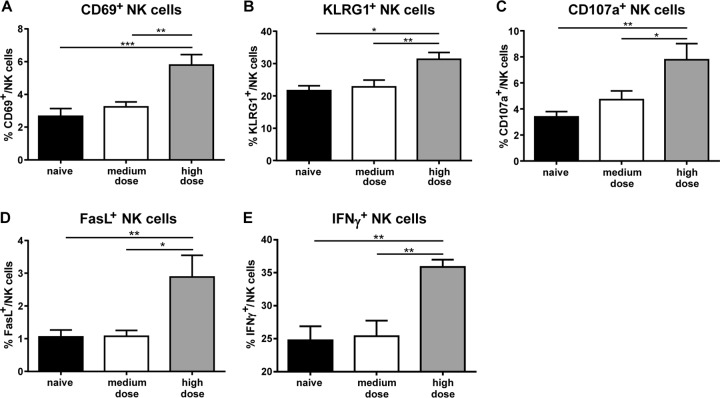
Phenotype of NK cells during an acute FV infection. Splenic NK cells from naive C57BL/6 mice or mice infected with 20,000 SFFU (medium dose) or 40,000 SFFU (high dose) of FV were analyzed by flow cytometry by gating on CD3^−^ NK1.1^+^ CD49b^+^ cells. Activation of NK cells was determined using the early activation marker CD69 (A). Maturated NK cells were identified by expression of KLRG1 (B). Effector functions were analyzed by expression of the degranulation marker CD107a (C) and FasL (D). The percentage of IFN-γ^+^ NK cells is shown in panel E. At least seven animals per group from at least three experiments were used for analysis. Mean values are shown, with standard errors of the means (SEM) indicated by error bars. Statistically significant differences between groups were analyzed with the Kruskal-Wallis test (A, B, and C) and ordinary one-way ANOVA (D and E) and are indicated as follows: *, *P* < 0.05; **, *P* < 0.01; and ***, *P* < 0.001.

In a previous study it was demonstrated that a low- or medium-dose FV infection did not result in a potent activation of NK cells with antiviral activity (41). To address the question of whether NK cell activation is dependent on the initial infection dose, we analyzed the NK cell phenotype in naive mice and mice infected with a medium or high dose of FV at 3 dpi. First the activation of NK cells (CD3^−^ NK1.1^+^ CD49b^+^) was determined by using the early activation marker CD69 (Fig. 2A). We detected no increase in the percentage of activated NK cells after low-dose (data not shown) or medium-dose infection compared with findings for naive mice, confirming our previous data. However, we found significantly higher frequencies of CD69^+^ NK cells in the group of mice infected with high virus doses than for both of the other groups. Very similar results were obtained for staining with the NK cell maturation marker KLRG1. Again, only high-dose infection induced maturation of NK cells (Fig. 2B). Furthermore, we analyzed the expression of the degranulation-associated molecule CD107a (lysosome-associated membrane protein 1 [LAMP-1]) and observed no significant increase in the percentage of CD107a-expressing NK cells in medium-dose-infected mice compared to that in naive mice (Fig. 2C). Remarkably, high-dose FV infection resulted in substantially increased percentages of CD107a^+^ NK cells in the spleen. We next analyzed the apoptosis-inducing FasL and again found that the frequency of FasL^+^ NK cells was enhanced only after high-dose virus inoculation (Fig. 2D). In viral infections, NK cell activation is often associated with the release of the proinflammatory and antiviral cytokine IFN-γ (5). We therefore determined the percentages of IFN-γ^+^ NK cells (Fig. 2E). Medium-dose FV infection did not result in increased percentages of IFN-γ^+^ NK cells compared to those in naive mice, whereas high-dose virus challenge led to a significant increase in the percentage of IFN-γ^+^ NK cells. To exclude the possibility that a medium-dose infection delayed the NK cell response, we analyzed the phenotype of NK cells at 5 dpi. We did not detect any increased activation (CD69^+^ and KLRG1^+^) and effector functions (CD107a^+^, FasL^+^, and IFN-γ^+^) of NK cells in medium-dose-infected mice at 5 dpi compared to those in uninfected mice or infected mice at 3 dpi (data not shown). Taken together, these results demonstrate that a medium-dose infection did not induce NK cell activation, maturation, or effector function at day 3 postinfection, whereas high virus inoculation resulted in a detectable NK cell response.

### Only high-dose FV infection induces NK cells that can kill target cells *in vitro* and *in vivo*.

**FIG 3:**
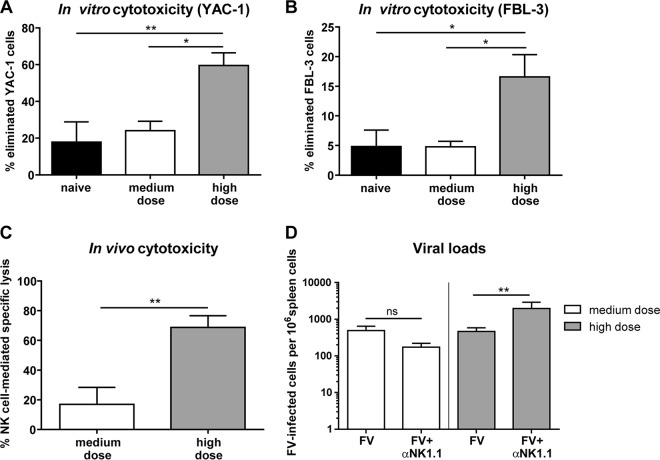
Cytotoxic functions of NK cells in medium- and high-dose FV infections. C57BL/6 mice were naive or infected with a medium dose (20,000 SFFU) or high dose (40,000 SFFU) of FV. Splenic NK cells were isolated and coincubated with CFSE-labeled YAC-1 cells (A) or FV-transformed tumor cells (FBL-3 cells [B]). Cells were stained for viability and immediately analyzed using flow cytometry. For panel C, mice received 5 × 10^5^ CFSE-labeled RMA/S and 5 × 10^5^ eFluor 670 cell tracer-labeled RMA cells at 3 dpi i.p. Two days later (5 dpi), cells were obtained by peritoneal lavage and analyzed by flow cytometry. Mean values are shown, with SEM indicated by error bars. Viral loads were analyzed in the spleen at 3 dpi by IC assay (D). NK cells were depleted with the NK1.1-specific monoclonal antibody PK136 (FV+αNK1.1). Statistically significant differences between groups were analyzed by the Kruskal-Wallis test (A and B) or Mann-Whitney test (C and D) and are indicated as follows; *, *P* < 0.05, and **, *P* < 0.01. At least five animals per group were examined. The experiments were repeated at least twice, with comparable results.

We demonstrated activation of NK cells in the group of mice that receive a high-dose virus inoculum, so we further addressed whether these NK cells were really able to kill potential target cells. First, we analyzed the cytotoxicity of NK cells *in vitro* by using the MHC-I-negative target cell line YAC-1 or the FV-transformed murine leukemia cell line FBL-3 (37, 60) and NK cells from mice at 3 dpi. Indeed, we found significantly augmented killing not only of YAC-1 cells (Fig. 3A) but also of FV-induced FBL-3 cells (Fig. 3B) in high-dose-FV-infected mice in comparison to that in medium-dose-infected or naive mice. We confirmed the *in vitro* cytotoxicity in an *in vivo* NK cell killing assay by injecting RMA control cells and MHC processing-defective RMA/S cells (60) into FV-infected mice (Fig. 3C). We observed a 4-fold-enhanced NK cell-mediated killing in recipient mice infected with the high virus dose. To address the question of whether the activation and cytotoxicity of NK cells constitute the reason for the efficient virus control in mice infected with the high dose of FV (already at day 3 postinfection no difference in viral loads was detected between the mice infected with a medium or high dose of virus, as shown in Fig. 1), we ablated NK cells to show their biological impact on viral replication. As expected, NK cell depletion in high-dose-infected mice significantly augmented viral loads (Fig. 3D). In contrast, no significant effect of NK cell ablation was found in animals that were inoculated with a medium dose of FV. These results indicate that NK cells can contribute to the control of acute retroviral infection *in vivo*, but only if they are fully activated due to high virus replication during initial infection.

### DCs and macrophages are highly activated in high-dose-FV-infected mice.

**FIG 4:**
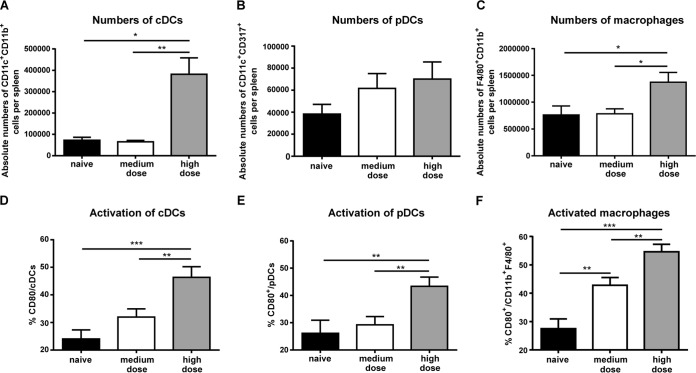
Absolute numbers and activation of DCs and macrophages during an acute FV infection. C57BL/6 mice were infected with the indicated doses of FV. Uninfected mice were used as a control. Absolute numbers of cDCs (CD11c^+^ CD11b^+^ [A]), pDCs (CD11c^+^ CD317^+^ [B]), and macrophages (lin^−^ F4/80^+^ CD11b^+^ [C]) in the spleen were analyzed by flow cytometry. Activation of innate cells was identified by the expression of CD80 molecules on the cell surface (D, E, and F). At least seven animals per group from at least three experiments were used for analysis. Mean values are shown, with SEM indicated by error bars. Statistically significant differences between groups were analyzed with the Kruskal-Wallis test (A and C) or ordinary one-way ANOVA (D, E, and F) and are indicated as follows: *, *P* < 0.05; **, *P* < 0.01; and ***, *P* < 0.001.

Because we found increased activation and antiviral function of NK cells in high-dose-FV-infected mice, we ask whether activated DCs and macrophages may have contributed to NK cell activation. DCs and macrophages are known to interact with NK cells and produce cytokines, which are necessary for the induction and maintenance of NK cell functions (13, 14, 28, 61, 62). Conversely, NK cell activity, especially IFN-γ production, promotes DC and macrophage activation (63, 64). Therefore, we first analyzed the absolute cell numbers of CD11c^+^ CD11b^+^ DCs (cDCs) (Fig. 4A), CD11c^+^ CD317^+^ DCs (pDCs) (Fig. 4B), and macrophages (lineage negative [lin^−^] F4/80^+^ CD11b^+^) (Fig. 4C) in the spleens of mice at 3 dpi. In mice infected with a medium dose of virus, we detected no significant changes in the absolute numbers for all three cell types in comparison to those in uninfected mice, whereas after high-dose infection, substantial increases of cDC and macrophage cell numbers were detectable. We next addressed whether DCs and macrophages became activated during FV infection. Indeed, the analysis of the surface molecule CD80, which is associated with activation and maturation of cDCs (Fig. 4D), pDCs (Fig. 4E), and macrophages (Fig. 4F), revealed a significant increase in the percentage of activated cells for all three populations after high-dose FV infection. After medium-dose infection, only the frequency of activated macrophages was increased. Taken together, these results demonstrate that concomitant with the activation of NK cells after high-dose retroviral challenge, expansion and activation of DCs and macrophages were found, which might be instrumental for the initiation and perpetuation of the NK cell response.

### Enhanced IL-15 and IL-18 mRNA levels and protein concentrations in high-dose-FV-infected mice.

**FIG 5:**
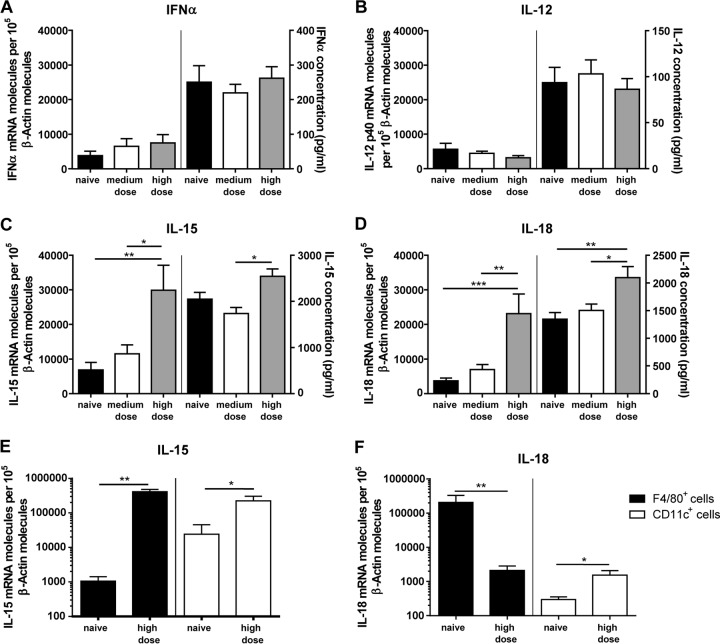
mRNA expression levels and protein concentrations of IFN-α, IL-12, IL-15, and IL-18 in naive mice and medium- and high-dose-infected mice. At 3 dpi, levels of IFN-α (A), IL-12 p40 (B), IL-15 (C), and IL-18 (D) mRNA expression were analyzed in the spleens of naive C57BL/6 mice or mice infected with a medium dose (20,000 SFFU) or high dose (40,000 SFFU) via quantitative real-time PCR (left-hand side of graphs). β-Actin was used as an internal standard and was amplified from each sample for normalization of the template concentration. Samples were run in duplicate and were analyzed by ordinary one-way ANOVA. At least four animals per group from at least three experiments were used for analysis. Spleens were harvested and single cell suspensions were prepared in a total of 1 ml. Cells were centrifuged and supernatants of splenic lavages were used for ELISAs (right-hand side). At least four animals per group from at least three experiments were used for analysis. F4/80^+^ macrophages or CD11c^+^ DCs were isolated from splenocytes using magnetic beads. The levels of IL-15 (E) and IL-18 (F) mRNA expression were analyzed via quantitative real-time PCR in F4/80^+^ and CD11c^+^ cells. β-Actin was used as an internal standard and was amplified from each sample for normalization of the template concentration. Samples were run in duplicate. At least three animals per group were used for analysis. Mean values are shown, with SEM indicated by error bars. Statistically significant differences between groups were analyzed with ordinary one-way ANOVA (A, C, and D), the Kruskal-Wallis test (B), or the Mann-Whitney test (E and F) and are indicated as follows: *, *P* < 0.05; **, *P* < 0.01; and ***, *P* < 0.001. Outliers were identified and removed with the Rout method.

As demonstrated above, NK cells were highly activated and effective during acute FV infection in mice that received high doses of the pathogen. NK cell maturation and activation require multiple cytokines, such as IL-12, IL-15, IL-18, and type I IFNs (5, 24, 64). We therefore addressed the question of whether we could find differences in the cytokine milieu after inoculation of different doses of FV. mRNA levels and the protein concentrations for IFN-α (Fig. 5A), IL-12 (Fig. 5B), IL-15 (Fig. 5C), and IL-18 (Fig. 5D) in naive, medium-dose-infected, and high-dose-infected mice were measured in spleens. Interestingly, we did not detect changes of IFN-α (Fig. 5A) or IL-12 (Fig. 5B) mRNA levels or protein concentrations after FV infection. However, the analysis of IL-15 showed a >4-fold increase of mRNA levels after high-dose viral infection compared to those in naive mice (Fig. 5C), whereas infection with a medium dose of FV did not result in upregulation of IL-15 mRNA expression. We also detected a significant increase in IL-15 protein concentration when comparing medium- and high-dose-FV-infected mice. For the expression of IL-18 we were able to show significant increases in mRNA and protein levels in high-dose-infected mice compared to those in naive mice and medium-dose-infected mice (Fig. 5D). To define the source of cytokines, we isolated F4/80^+^ macrophages and CD11c^+^ DCs from spleens of naive and high-dose-infected mice and measured the mRNA levels of IL-15 (Fig. 5E) and IL-18 (Fig. 5F) within these cell populations. We detected a strong increase in the IL-15 mRNA level in F4/80^+^ macrophages, but also an enhanced IL-15 mRNA level in the CD11c^+^ population of high-dose-infected mice. In high-dose-FV-infected mice, we found a reduction of the IL-18 mRNA level in the F4/80^+^ population but identified an increased IL-18 mRNA level in CD11c^+^ cells, suggesting that IL-18 is produced mainly by CD11c^+^ DCs. Thus, the cytokines IL-15 and IL-18, which are produced by macrophages and DCs (65, 66), might be the essential signals for the induction of the NK cell response, whereas the cytokines IFN-α and IL-12 are likely not involved.

### Neutralization of IL-15 and IL-18 results in reduced NK cell functions, whereas IL-15 and IL-18 therapy improve the NK cell response during acute FV infection.

**FIG 6:**
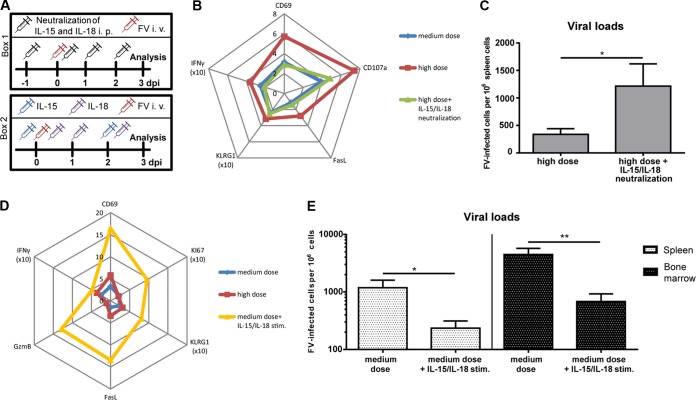
Effects of IL-15 and IL-18 on NK cell functions and viral loads. C57BL/6 mice were infected with 20,000 SFFU (medium dose) or 40,000 SFFU (high dose) of FV at day 0. For neutralization of IL-15 and IL-18, mice were treated as displayed in panel A, box 1. Therapy with IL-15 and IL-18 was performed as indicated in panel A, box 2. A spider plot from mean percentages of NK cell activation (CD69 [*P* = 0.0135]), maturation (KLRG1 [*P* = 0.0476]), degranulation (CD107a [*P* = 0.0156]), and FasL (*P* = 0.0303) and IFN-γ (*P* = 0.0001) expression in infected mice (medium and high doses) and high-dose-infected mice with neutralization of IL-15 and IL-18 is shown in panel B. Viral loads of mice infected with 40,000 SFFU and with cytokine neutralization were analyzed by IC assay. At least four animals per group from two experiments were used for the analysis. Mean values are shown, with SEM indicated by error bars. Statistically significant differences between groups were analyzed by the Mann-Whitney test (*, *P* < 0.05). A spider plot of NK cell activation (CD69 [*P* = 0.0001]) and functions (FasL [*P* = 0.0003], granzyme B [*P* = 0.0061], and IFN-γ [*P* = 0.0001]) as well as maturation (KLRG1 [*P* = 0.0001]) and proliferation (KI67 [*P* = 0.0061]) after IL-15 and IL-18 therapy is shown in panel D. At least six animals per group from two independent experiments were used. Statistically significant differences between the group of medium-dose-infected mice and medium-dose-infected and cytokine-treated mice were analyzed by theMann-Whitney test. Viral loads in spleens and the bone marrow of medium-dose-infected and medium-dose-infected and IL-15- and IL-18-treated mice are displayed in panel E. At least six animals per group from two independent experiments were used. Statistically significant differences between groups were analyzed by the Mann-Whitney test and are indicated as follows: *, *P* < 0.05, and **, *P* < 0.01.

To prove that the cytokines IL-15 and IL-18 contribute to the cytotoxic functions of NK cells after high-dose FV challenge, we neutralized both cytokines during infection (Fig. 6A, box 1). At 3 dpi, NK cell activation (CD69), maturation (KLRG1), IFN-γ production, and cytotoxic effector functions such as degranulation (CD107a) and FasL expression were analyzed; these are demonstrated in a spider plot (Fig. 6B). Whereas the percentages of positive NK cells from high-dose-infected mice were significantly higher for all five markers than for animals infected with a medium dose, the neutralization of IL-15 and IL-18 in the high-dose group completely abrogated this activation of NK cells. After cytokine neutralization the NK cells were indistinguishable from those of medium-dose-infected or naive mice (data not shown). Besides the analysis of NK cells, we also determined the effect of cytokine blockage on viral loads (Fig. 6C). An almost 4-fold increase in viral set points in high-dose-FV-infected mice with IL-15 and IL-18 neutralized in comparison to those in high-dose-infected mice was found. To test whether the NK cell response can be therapeutically augmented with the cytokines IL-15 and IL-18, we treated medium-dose-infected mice with these cytokines, which do usually not show NK cell activation (Fig. 6A, box 2). Cytokine treatment significantly enhanced the percentages of NK cells that were activated (CD69^+^), proliferating (KI67^+^), and mature (KLRG1^+^). We also detected higher percentages of effector NK cells, measured by FasL and granzyme B (GzmB) expression. After cytokine stimulation, percentages of IFN-γ^+^ NK cells were also increased in comparison to those in untreated mice. To analyze the therapeutic effect of the exogenous IL-15 and IL-18 therapy, we measured the viral loads in treated and untreated animals that were infected with a medium dose of FV (Fig. 6E). Indeed, we detected a significant decrease in viral burden after administration of the cytokines IL-15 and IL-18 in spleens. This therapeutic effect was more pronounced in another target organ, the bone marrow. Taken together, our data reveal the importance of IL-15 and IL-18 in the initiation of an efficient antiretroviral NK cell response and demonstrate a therapeutic benefit of IL-15 and IL-18 therapy during retroviral infection.

## DISCUSSION

Despite recent success in antiretroviral therapy, curing HIV infection is still impossible. To get deeper insights into host defense mechanisms and to find potential targets for antiviral immunotherapies, research on retroviral immunity is indispensable. In this study, we analyzed the impact of the infection dose on the initiation of an NK cell response. Our data clearly demonstrate the importance of high-level virus replication in triggering antiretroviral NK cell activity.

Until now, not much has been known about the immunobiology of infections with different viral doses or how this parameter influences the induction of the NK cell activity. Researchers have analyzed the effect of different viral doses in LCMV infection (34, 67) using LCMV clone 13 as a well-established model of persistent infection (68). In one of these studies, virus replication was controlled early in low-dose-infected mice, which correlated with an early CD4^+^ T cell response followed by CD8^+^ T cell expansion. Both medium- and high-dose infections resulted in loss of body weight, but medium-dose infection resulted in accelerated wasting, lung pathology, and morbidity (67). Waggoner and colleagues described an important role of NK cells in mice infected with different LCMV doses (34). With an intermediate LCMV dose, NK cells suppressed antiviral T cells and depletion of NK cells restored T cell responses, resulting in the survival of animals. In contrast, NK cell-mediated suppression of T cell responses after high-dose LCMV infection was beneficial, and depletion of NK cells was followed by immunopathology and mortality (34). However, in these studies only indirect effects of NK cells on virus infection via modulating T cell responses were investigated. Accordingly, these studies were performed at later time points of infection, when T cell responses can be detected. Our study instead focused on direct antiviral effects of NK cells on viral loads before T cells become active. During acute FV infection, virus-specific CD4^+^ and CD8^+^ T cells were detectable at day 10 postinfection, but no activation was detected at 7 dpi (39, 49). Furthermore, T cell numbers, proliferation (KI67), maturation (KLRG1), and activity (CD69 and CD107a) were not affected by medium or high virus dose at 7 dpi (data not shown). Thus, during early acute FV infection (3 dpi), the viral loads after high-dose virus inoculation were directly controlled by NK cells, as demonstrated in Fig. 1 and 3D. In 2016, Gillespie and colleagues analyzed genetic factors that control viral spread during mouse cytomegalovirus (MCMV) infection among different strains of mice, and they showed for mice of an MCMV-resistant strain infected with a high dose of MCMV increased frequencies of NK1.1^+^ NKp46^+^ NK cells and corresponding lower viral loads (69). They concluded that NK cell frequencies and NKp46^+^ NK cells in MCMV infection depend on distinct loci that regulate NK cells and host resistance (69). While we also demonstrated highly active NK cells in high-dose infection corresponding with reduced viral loads, the frequency of NK cells in FV-infected mice was not dependent on the infection dose (data not shown). Thus, our data show that NK cells are necessary regulators of retroviral infection during high-dose but not during low- and medium-dose infections and depend predominantly on a certain level of virus particles that initiate an effective immune response. In the influenza virus mouse model the importance of infection doses on the NK cell response has also been shown. In experiments with low-level influenza virus infection, depletion or inhibition of NK cells resulted in severe morbidity and mortality (70, 71), whereas in severe influenza virus infection, NK cells mediated enhanced immunopathology (33). Hence, in high-dose influenza virus infection, the depletion of NK cells resulted in improved survival and recovery of mice (33). In the current study, NK cell depletion during acute FV infection led to no significant changes after medium-dose infection, whereas in high-dose infection, depletion increased viral loads (Fig. 3D). Thus, activated and effective NK cells were required for the control of severe retroviral infection.

**FIG 7:**
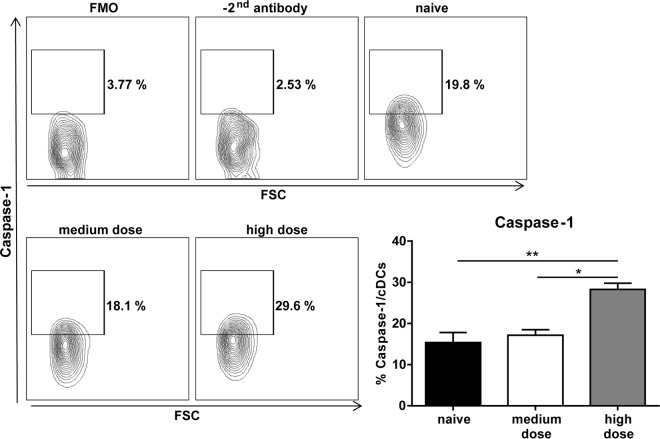
Caspase 1 activity in cDCs. C57BL/6 mice were infected with 20,000 SFFU (medium dose) or 40,000 SFFU (high dose) of FV. Naive mice were used as a control. Caspase 1 activity was analyzed in cDCs using flow cytometry. At least five animals per group from at least three experiments were used for the analysis. Mean values are shown, with SEM indicated by error bars. Statistically significant differences between groups were analyzed by the Kruskal-Wallis test and are indicated as follows: *, *P* < 0.05, and **, *P* < 0.01. FMO, fluorescence minus one; FSC, forward scatter.

Activation of NK cells depends on a variety of factors, such as contact-dependent stimulation and cytokines. Cytokine treatment for inducing antiviral or anticancer immune responses has been reported in many studies (72–77). However, immunotherapy with cytokines such as IL-2 stimulated not only cytotoxic immune cells but also suppressive regulatory T cells (Tregs) (78). In contrast, the cytokine IL-15 shares many similarities with IL-2 but stimulates mainly CD8^+^ T cells and NK cells rather than Tregs (79). Interestingly, IL-15 is constitutively expressed on the surface of macrophages and DCs, and IFN-γ can further upregulate the expression of IL-15 to improve NK cell development, survival, and activity (80, 81). During acute FV infection we found increased numbers of activated DCs and macrophages in high-dose-infected mice (Fig. 4A and C), which correlated with enhanced IFN-γ production and activation of NK cells (Fig. 2). Also, adaptive immune cells, such as CD8^+^ T cells, require IL-15 for their development (82, 83), but during the initial phase of acute FV infection (3 dpi), T cell responses are not detectable (39, 49). IL-15 has been postulated to play an important role in immunity in HIV-1 infection, but results published so far have been contradictory. On one hand, a positive correlation between IL-15 levels and viremia in HIV patients was described (84, 85). On the other hand, IL-15 was reported to enhance the anti-HIV function of immune cells (86, 87). Furthermore, immunotherapeutic success in HIV-1-infected humanized mice was achieved with a superagonistic IL-15 antibody (72). In that study, increased antiviral activity of NK cells and a reduction in acute HIV-1 viral loads were described (72). Besides the beneficial role of IL-15 in viral infections, application of an IL-15 fusion protein significantly reduced tumor growth and progression in a melanoma mouse model, which was correlated with enhanced NK cell activity (73). During acute high-dose FV infection, IL-15 production was induced, suggesting that strong virus replication can trigger IL-15, most likely due to the activation of macrophages (Fig. 4F) and cDCs (Fig. 4D) that sense virus infection. Both cell types can be infected with FV (88, 89), but whether productive infection is required for IL-15 induction remains elusive. Besides elevated levels of IL-15, we also detected production of IL-18 during high-dose FV infection (Fig. 5D). Interestingly, infections with HIV or hepatitis C virus (HCV) are also associated with high levels of plasma IL-18 (90, 91). The immunostimulatory cytokine IL-18 acts synergistically together with IL-15 in stimulating natural killer cells and is known to promote IFN-γ production by NK cells (92–94). We also found a correlation between IL-18 induction and IFN-γ production by NK cells, which was abrogated by IL-18 neutralization (Fig. 6B). Furthermore, a significant increase of viral loads was detectable after IL-15 and IL-18 neutralization, elucidating the importance of these cytokines in retroviral immunity (Fig. 5C). During infections, IL-18 can be secreted by DCs and macrophages (95, 96), which were also highly activated during high-dose FV infection (Fig. 4D to F). IL-18 is constitutively released in an inactive pro-IL-18 form and requires further processing by the intracellular cysteine caspase 1 to become bioactive (93). The proinflammatory caspase 1 is recruited and activated by multimeric protein complexes termed inflammasomes. Inflammasome activation results in inflammation, modulation of the adaptive immune system, and direct antiviral effects (97–99). Our data suggest that severe FV infection is sensed by inflammasomes and that this initial step might be very important for full activation of antiviral NK cells, which is supported by the finding that the IL-18 processing enzyme caspase 1 is also significantly upregulated in high-dose-infected mice but not in naive and medium-dose-infected mice (Fig. 7). Interestingly, other viral infections, such as infections with HIV and HCV, also activate the inflammasome pathway (100). In contrast, type I interferons do not seem to play a major role in NK cell activation during FV infection, which is in line with our previous findings (41, 101) and might be explained by a direct suppression of interferon responses by murine retroviruses (102). Not surprisingly, potent NK cell responses can be induced in FV-infected mice by injecting exogenous IFN-α, especially IFN-α subtypes 1 and 11 (41). NK cell proliferation as well as IL-15 and IL-18 release by macrophages and DCs occurs early during infections (103). IL-15 is critical for NK cell development survival and homeostasis (83, 104, 105). IL-18, in contrast to IL-15, does not influence the development or homeostasis of NK cells (106, 107), but French and colleagues demonstrated that the IL-18 receptor is constitutively expressed on splenic NK cells and IL-18 contributes to NK cell proliferation during infections (94). Neutralization of IL-15 and IL-18 in mice acutely infected with high-dose FV resulted in a substantial increase in viral burden correlating with the loss of NK cell functions. Furthermore, IL-15 and IL-18 therapy improved NK cell responses in medium-dose-infected mice. These data demonstrate the importance of cytokines for NK cell activation in retroviral infections and may suggest that immunotherapy combining the well-tolerated cytokines IL-15 and IL-18 might be an interesting new approach for antiretroviral treatment.

## MATERIALS AND METHODS

### Mice and virus.

Animal experiments were performed in strict accordance with the German regulations of the Society for Laboratory Animal Science (GV-SOLAS) and the European Health Law of the Federation of Laboratory Animal Science Associations (FELASA). Experiments were done using female inbred C57BL/6 mice (Harlan Laboratories, Germany) that were between 7 and 10 weeks old at day 0 of the experiment. For the experiments, an FV complex containing B-tropic Friend murine leukemia helper virus and polycythemia-inducing spleen focus-forming virus was used. The FV stock was prepared as a 15% spleen cell homogenate from BALB/c mice infected 14 days previously with 3,000 SFFU of FV. Mice were injected intravenously with 0.15 ml of phosphate-buffered saline (PBS) containing 5,000 SFFU (low dose), 20,000 SFFU (medium dose), or 40,000 SFFU (high dose) of FV. By injection of 20,000 or 40,000 SFFU of FV, mice received 20,000 or 40,000 infectious virus particles that can induce the proliferation of spleen cells *in vivo*. A 2-fold increase in virus titers has shown to be of major importance for the outcome of infection *in vivo*. The virus stock was free of lactate dehydrogenase-elevating virus. Mice were sacrificed at 1 dpi, 3 dpi, or 5 dpi by cervical dislocation.

### Infectious-center assay.

For the detection of infectious centers, 10-fold dilutions of single-cell suspensions onto Mus dunnis cells were prepared. Cultures were incubated for 3 days, then fixed with ethanol, stained with the F-MuLV envelope-specific monoclonal antibody 720, and developed with a peroxidase-conjugated goat anti-mouse antibody and aminoethylcarbazol for the detection of foci.

### Flow cytometry.

Surface stainings were done using antibodies against the following: CD3 (17A2), CD4 (RM4-5), CD8 (53-6.7), CD11b (M1/70), CD11c (N418), CD49b (DX5), CD80 (16-10A1), CD107a (ID4B), CD317 (927), F4/80 (BM8), KLRG1 (2F1), and NK1.1 (PK136). For the exclusion of dead cells, splenocytes were stained with a Zombie UV fixable viability kit (BioLegend). Gating of the lineage-negative (lin^−^) population was performed by exclusion of singlets, dead cells, T cells, and NK cells. After surface staining, granzyme B (GB11) was stained intracellularly after fixation with BD Cytofix/Cytoperm. Cells were fixed with the Foxp3/transcription factor staining buffer set (eBioscience) to analyze the expression of KI67 (B56). For IFN-γ (XMG1.2) and FasL (MFL3) restimulation, cells were restimulated with ionomycin (500 ng/ml), phorbol myristate acetate (PMA; 25 ng/ml), monensin (1×), and brefeldin A (2 μg/ml) diluted in Iscove's modified Dulbecco's medium (IMDM) buffer and incubated for 3 h at 37°C. Splenocytes were fixed with a BD Cytofix/Cytoperm fixation/permeabilization kit. For the detection of caspase 1 activity, splenocytes were stained for surface markers and fixed with the BD Cytofix/Cytoperm kit. After fixation, monoclonal IgG1 caspase 1 antibody (Santa Cruz Biotechnology) was added for 30 min. After a washing, cells were incubated for 30 min with an allophycocyanin-coupled anti-IgG1 antibody (BD Pharmingen).

### *In vitro* cytotoxicity assay.

NK cell cytotoxicity was tested *in vitro* using 1 × 10^4^ carboxyfluorescein succinimidyl ester (CFSE)-stained YAC-1 or FBL-3 tumor cells and 25 × 10^4^ isolated NK cells from the spleens of naive or FV-infected mice. NK cells were isolated using a MagniSort mouse NK cell enrichment kit (eBioscience). The cytotoxic assay was performed in 96-well U-bottom plates. The cells were coincubated for 24 h in a humidified 5% CO_2_ atmosphere at 37°C. Cells were washed once and stained with fixable viability dye (eBioscience) to exclude dead cells. After a washing, cells were directly analyzed by flow cytometry.

### *In vivo* cytotoxicity assay.

An *in vivo* NK cell cytotoxicity assay was executed using 5 × 10^5^ RMA/S cells labeled with 10 μM CFSE (Vybrant CFDA SE cell tracer kit; Life Technologies) and 5 × 10^5^ RMA cells labeled with a 2.5 μM concentration of the cell proliferation dye eFluor 670 (eBioscience) per mouse. Cells were mixed in a ratio of 1:1 and injected intraperitoneally (i.p.) at 3 dpi. NK cell-depleted mice were used as negative controls. Mice were sacrificed at 5 dpi, and intraperitoneal lavage was performed with 10 ml of PBS to obtain cells. Cells were washed once, resuspended in buffer containing fixable viability dye (eBioscience) for the exclusion of dead cells, and analyzed with a flow cytometer. The ratio of CFSE-labeled target cells (RMA/S) versus eFluor 670-labeled nontarget cells (RMA) was calculated normalized to the ratio in NK cell-depleted animals.

### Depletion of NK cells.

NK cells were ablated by intraperitoneal injection of 0.2 ml of supernatant fluid containing NK1.1-specific monoclonal antibody PK136. For depletion of NK cells, depletion antibody was injected 1 day prior to and 2 days after FV infection. More than 90% of NK1.1^+^ cells were depleted.

### ELISA.

Murine spleens were harvested and grinded using 70-μm strainers. Cells were collected in 1 ml of PBS. After centrifugation, supernatants of splenocytes were used for enzyme-linked immunosorbent assays (ELISAs). ELISAs were performed according to the manufacturer's instructions with a mouse IFN-α (multiple subtypes) bioluminescent ELISA kit (InvivoGen), IL-12 p70 ELISA MAX Deluxe (BioLegend), mouse IL-15 ELISA Ready-SET-Go! (eBioscience), and mouse IL-18 Platinum ELISA (eBioscience).

### RNA isolation.

Total RNA was isolated from splenocytes and F4/80^+^ (phycoerythrin [PE]; clone BM8) or CD11c^+^ (PE; clone N418) cells with anti-PE microbeads (Miltenyi Biotec), utilizing DNA/RNA Shield (Zymoresearch) and an innuPREP RNA minikit (Analytik Jena). cDNA was synthesized using innuScript reverse transcriptase (Analytik Jena). cDNA was stored at −20°C.

### Real-time PCR.

Real time-PCR analysis for the quantification of IFN-α, IL-12, IL-15, and IL-18 mRNA was performed using innuMIX quantitative PCR (qPCR) MasterMix SyGreen (Analytik Jena). Oligonucleotide sequences (Biomers) for tested genes were as follows: for β-actin, 5′-AAATCGTGCGTGACATCAAA-3′ and 5′-CAAGAAGGAAGGCTGGAAAA-3′; for IFN-α, 5′-ATGGCTAGGCTCTGTGCTTTCC-3′ and 5′-AGGGCTCTCCAGACTTCTGCTCTG-3′; for IL-12 p40, 5′-ACAGCACCAGCTTCTTCATCAG-3′ and 5′-TCTTCAAAGGCTTCATCTGCAA-3′; and for IL-15, 5′-CATTTTGGGCTGTGTCAGTG-3′ and 5′-TCTTCAAAGGCTTCATCTGCAA-3′. The Mm-Il18-1-SG QuantiTect primer assay (Qiagen) was used. The quantitative mRNA levels were determined by using Rotor-Gene Q series software (Qiagen) and were normalized to β-actin mRNA expression levels.

### Neutralization of the cytokines IL-15 and IL-18.

Mice were injected every day i.p. with IL-15-neutralizing (75 μg, anti-mouse IL-15 functional grade purified; Bioscience) and IL-18-neutralizing (200 μg, InVivoMAb anti-mouse IL-18; BioXCell) antibodies starting 1 day before high-dose FV infection.

### IL-15 and IL-18 stimulation.

Mice were infected with a medium dose of FV. Starting from day 0, mice were injected i.p. twice with IL-15 (2.5 μg, recombinant mouse IL-15; BioLegend) and three times with IL-18 (1 μg, recombinant mouse IL-18; R&D Systems).

### Statistical analyses.

Statistical analyses and graphical presentations were done with GraphPad Prism version 6 and Excel 2010. Statistical differences between two different groups were determined by the Mann-Whitney test. Analyses including several groups were tested using the nonparametric Kruskal-Wallis one-way analysis of variance (ANOVA) on ranks and Dunn's multiple-comparison test or ordinary one-way ANOVA and Tukey multiple-comparison test.
